# FUNSAT: A FUNCTIONAL SKILLS TRAINING PROGRAM

**DOI:** 10.1093/geroni/igad104.1299

**Published:** 2023-12-21

**Authors:** Sara Czaja, Philip Harvey, Peter Kallestrup

**Affiliations:** Weill Cornell Medicine, New York City, New York, United States; University of Miami Miller School of Medicine, Miami, Florida, United States; i-Function, Miami, Florida, United States

## Abstract

Performance of everyday activities is often challenging for older adults, especially for aging adults with a cognitive impairment (CI), which threatens independence and quality of life. We developed a novel computer-based functional skills assessment and training (FUNSAT) program, which includes simulations of everyday technology-based tasks (e.g., online banking, prescription refill, online shopping) important to everyday living. This presentation will present preliminary findings from our study that is evaluating the FUNSAT program with aging adults with and without a cognitive impairment. The sample includes 153 adults (83 with a CI, 130 females and 23 males) ranging in age from 60-92 yrs. Initially, participants completed the FUNSAT assessment and then engaged in the FUNSAT training program for up to 24 hours over 12 weeks at home after an initial in-person training. The non-cognitively impaired participants completed the FUNSAT training alone. The participants with a cognitive impairment were randomized into one of two conditions; FUNSAT training alone, or Cognitive Training (3 weeks) followed by FUNSAT training. We also evaluated transfer of training using ecological momentary assessment (EMA). Our preliminary findings indicate that participants irrespective of cognitive status are demonstrating significant gains in performance following training on the training tasks. Further, they are reporting an increase in confidence with respect to performing the tasks in daily living and the EMA data suggest transfer of training to other technology-based tasks. We will also discuss adherence to the home training protocol, which has important implications for implementation of the program beyond the research setting.

